# Charpy Impact Behavior of a Novel Stainless Steel Powder Wire Mesh Composite Porous Plate

**DOI:** 10.3390/ma14112924

**Published:** 2021-05-28

**Authors:** Chaozhong Li, Zhaoyao Zhou

**Affiliations:** National Engineering Research Center of Near-Net-Shape Forming for Metallic Materials, School of Mechanical and Automotive Engineering, South China University of Technology, Guangzhou 510640, China; melichaozhong@mail.scut.edu.cn

**Keywords:** powder wire mesh composite porous plate, impact toughness, 304 stainless steel, rolling, solid phase sintering

## Abstract

A novel powder wire mesh composite porous plate (PWMCPP) was fabricated with 304 stainless steel powders and wire mesh as raw materials by vacuum solid-state sintering process using self-developed composite rolling mill of powder and wire mesh. The effects of different mesh volume fractions, mesh diameters, and sintering temperatures on the pore structure and Charpy impact properties of PWMCPPs were studied. The results show that PWMCPPs have different shapes and sizes of micropores. Impact toughness of PWMCPPs decreases with increasing wire mesh volume fraction, and increases first and then decreases with increasing wire mesh diameter, and increases with increasing sintering temperature. Among them, the sintering temperature has the most obvious effect on the impact toughness of PWMCPPs, when the sintering temperature increased from 1160 °C to 1360 °C, the impact toughness increased from 39.54 J/cm^2^ to 72.95 J/cm^2^, with an increased ratio of 84.5%. The tearing between layers, the fracture of the metallurgical junction, and the fracture of wire mesh are the main mechanisms of impact fractures of the novel PWMCPPs.

## 1. Introduction

Porous metal materials are composed of a metal frame structure and internal pores. They have the advantages of low density, high temperature resistance, controllable pore structure, high specific strength, large specific surface area, and machinability [1]. They are widely used in filtration [2,3,4], heat dissipation [5,6,7], noise reduction [8,9,10,11], medical treatment [12,13,14], catalysis [15,16,17,18], and other fields. With the continuous development of porous metal materials, they are not only used as an excellent functional material, but also as a structural material in the situation of complex stress, especially in the situation of impact load, such as the energy absorption and anticollision structure in the automotive field [19], which puts forward higher requirements for the impact performance of materials. Metal powder and wire mesh composites have broad application prospects because of the dual advantages of the powder microporosity and high strength of wire mesh.

In recent years, many scholars have carried out extensive research on the mechanical properties of porous metal materials. G. Straffelini et al. [20] carried out instrument impact tests of different samples of Fe–Mo–C high-strength steel with porous structure produced by the powder metallurgy method, and the impact fracture behavior of different samples of Fe–Mo–C high strength steel was investigated by measuring notch severity and matrix strength. The results show that with the increase of notch severity or matrix strength, the fracture modes range from ductile fracture to a very localized type of fracture, and from a very localized to a mixed type of fracture. They found that the pores on the surface of the material act as crack precursors. The impact toughness limit of the specimen with 2 mm opening depth reached 7.9 J/cm^2^. P. Liu et al. [21] studied the Charpy impact property and failure mechanism of the entangled steel wire porous metal material. It was found that the impact toughness of the material increases with decreasing porosity. The entangled steel wire porous metal material has excellent energy absorption performance, and its impact toughness limit value was 45.5 J/cm^2^. Ying Zhao et al. [22] used the drop hammer test machine to study the energy absorption and the mechanisms of deformation and damage of aluminum foam sandwich structures under the condition of low-velocity impact experiments. They found that the height and density of the core plate and the thickness of the panel had a significant effect on the impact resistance of the sandwich structure. The thicker panel and the better stiffness sandwich structure can resist higher impact load. M. W. Wu et al. [23] explored the effect of specific alloying elements on the microstructure and the impact toughness of powder metal steel. Experimental results show that Mo decreased the impact toughness of binary Fe–C alloys, but it increased the tensile strength. However, Ni and Cr not only increased the toughness, but also the tensile strength; their research results also confirmed that the increase of sintering temperature led to the enlarged sintered necks and the roundness of pores between powders, thus effectively improved the impact properties of powder metal steels. Shuiping Zou et al. [24] developed a new type of porous metal fiber powder sintered composite sheet by pressing and sintering metal fiber and copper powders with approximately spherical particles. It was demonstrated that the tensile strength of porous metal fiber powder sintered composite sheet decreases with increasing of porosity and increases with increasing of copper powder volume fraction. In addition, the increase of sintering temperature was able to significantly enhance the tensile strength of the material. Bibo Yao et al. [25] prepared a new type of 304 stainless steel powders metallurgy material with 304 stainless steel powders and 304 stainless steel short fibers as raw materials by pressing and vacuum solid-state sintering. The effects of fiber volume fraction, pressing force, and high-temperature nitriding on the compressive properties of the material were studied. The experimental results indicated that higher compression force and fiber volume fraction enhanced the compressive properties of the material, and the high-temperature nitriding was able to increase the elastic deformation stage of the material, and significantly improved the compressive strength. Zhaoyao Zhou et al. [26] prepared a novel stainless steel porous twisted wire material by cutting stainless steel wire ropes with self-developed rotary multi cutter tool. The porous structure and Charpy impact property of stainless steel porous twisted wire material were studied. They indicated that the impact toughness of stainless steel porous twisted wire material decreases with increasing of porosity, and the limit value of impact toughness was 17.9 J/cm^2^. However, metal powder and wire mesh composites have rarely been studied directly. The low mechanical properties, especially the impact properties, seem to be a common problem in porous metal materials. Therefore, it is very important to find a new processing method and explore processing parameters for improving the impact properties of porous metal materials.

In this work, a novel stainless steel powder wire mesh composite porous plate (PWMCPP) was developed by the processing method of composite rolling and solid-state sintering. The effects of the wire mesh volume fraction, wire mesh diameter, and sintering temperature on the Charpy impact properties of PWMCPPs were studied. The fracture mechanism of PWMCPPs was determined by observing the fracture morphology with scanning electron microscope.

## 2. Materials and Methods

### 2.1. Manufacturing Processes of the PWMCPPs

The materials used in the experiment were 304 stainless steel powders with an average size of 50–90 μm, and 304 stainless steel wire meshes with diameters of 40 μm, 50 μm, 60 μm, 70 μm, 80 μm, 100 μm, and 120 μm, respectively.

The manufacturing process of PWMCPP was mainly divided into five steps. First, powders and wire meshes (diameter of 40 μm) were combined and rolled into powder wire mesh composite porous strip (PWMCPS) by a self-developed rolling mill (roll diameter of 122 mm, rolling force of 50 t, rolling gap of 0 mm, and rolling speed of 1 mm/s). Then, the PWMCPS was annealed in a vacuum sintering furnace (WHS-20). The heating process was as follows: the PWMCPS was heated to 900 °C at a heating rate of 10 °C per minute at approximately 1×10^−4^ Pa, held for 2 h, and then cooled to room temperature. Then, the annealed PWMCPS was cut into powder wire mesh composite porous thin plate (PWMCPTP). Then, PWMCPPs with different wire mesh volume fractions (12.45%, 27%, 41.57%, 56.18%, and 70.81%) and different wire mesh diameters (50 μm, 60 μm, 70 μm, 80 μm, and 100 μm) were fabricated by compound rolling of PWMCPTP and stainless steel wire mesh with different layers and diameters by large rolling mill (roll diameter of 400 mm, rolling force of 240 t, rolling gap of 0 mm, and rolling speed of 340 mm/s). The volume fraction of wire mesh in the PWMCPP was calculated by Equation (1).

(1) $$\eta =\frac{n\times m_{1}}{m_{2}}\times 100\%.$$

where *η* is the volume fraction of wire mesh, *n* is the number of layers of wire mesh, *m*_1_ is the mass of one layer of wire mesh (g), and *m*_2_ is the mass of the PWMCPP (g).

**Figure 1:**
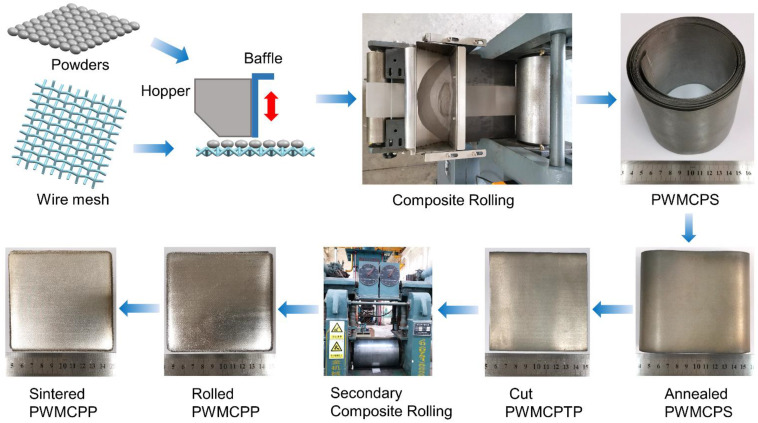
Manufacturing processes of the PWMCPPs.

Among them, PWMCPPs with different sintering temperatures were made of wire mesh with the diameter of 120 μm. Finally, the rolled PWMCPP was made into sintered PWMCPP by vacuum solid-phase sintering in a vacuum sintering furnace (WHS-20). The heating process was as follows: first, the PWMCPP was heated to 800 °C at a heating rate of 5 °C/min, and then heated to the highest temperature at a heating rate of 4 °C/min. Among them, PWMCPPs with different wire mesh volume fractions and wire mesh diameters were sintered at 1310 °C. PWMCPPs were sintered at different temperatures of 1160 °C, 1210 °C, 1260 °C, 1310 °C, and 1360 °C, respectively, held for 2 h, and then the temperature was reduced to 800 °C at a cooling rate of 5 °C/min. Finally, the PWMCPPs were cooled to room temperature. The manufacturing process of the PWMCPPs is shown in Figure 1.

In order to reflect the differences clearly among three groups of specimens: with different wire mesh volume fractions, with different wire mesh diameters, and with different sintering temperatures, the processing parameters of different PWMCPPs are summarized in Table 1.

### 2.2. Surface Morphology and Charpy Impact Characterization of the PWMCPPs

The surface morphology of four kinds of PWMCPPs was observed by a scanning electron microscope (Quanta 200, FEI, Eindhoven, The Netherlands). Four kinds of PWMCPPs were observed: volume fraction of 12.45%, diameter of 50 μm, and sintering temperatures of 1160 °C and 1310 °C. Charpy impact specimens were made with PWMCPPs parallel to the rolling direction. Specimens were tested in a pendulum impact testing machine (ZBC2302-2, Suns, Shenzhen, China). The size of the specimens with notch was 55 × 10 × *h* mm^3^ (where *h* is the thickness of PWMCPPs (mm)). The diameter of the bottom of V-notch was 0.25 mm, the depth was 2 mm, and the opening angle was 45°. A pendulum with an energy of 50 J was lifted up at an Angle of 150.21°. The span of the testing machine was 40 mm, and the impact absorption energy of the sample was directly read from the testing machine. Because the thickness of the sample was not exactly consistent, gaskets of appropriate thickness were added under the sample to make the center of the sample always coincide with the center of the pendulum, so as to ensure the accuracy of the impact absorption energy measurement. Before the impact test, the porosity of the PWMCPP was calculated according to Equation (2).

(2) $$P=\left(1-\frac{m}{\rho v}\right)\times 100\%$$

where *m* is the mass of the sample (g), *v* is the volume of the sample, and *ρ* is the density of 304 stainless steel (7.93 g/cm^3^).

The value of impact toughness as the energy required to achieve fracture per unit area was calculated according to Equation (3).

(3) $$\alpha_{k}=\frac{W_{k}}{A}$$

where *W_k_* is the absorbing energy (J), *A* is the cross-section at the breakdown (cm^2^), and *α_k_* is the impact toughness (J/cm^2^).

Three specimens of each parameter were used for the test, and the average value was taken as the test result. The fracture morphology of the specimen after impact was observed by scanning electron microscopy (Quanta 200, FEI, Eindhoven, The Netherlands), and the deformation and failure mechanism of the samples were analyzed.

## 3. Results and Discussion

### 3.1. Surface Morphology of Novel PWMCPPs

**Figure 2:**
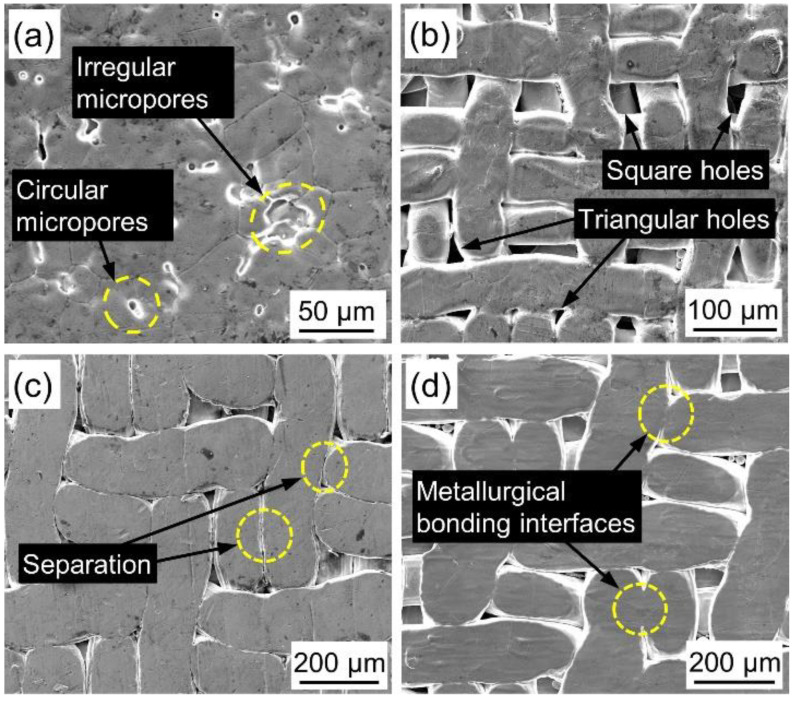
Surface topography of PWMCPPs. With (**a**) 12.45% volume fraction; (**b**) 50 μm wire mesh diameter; (**c**) 1160 °C sintering temperature; (**d**) 1360 °C sintering temperature.

Figure 2 shows the surface morphology of PWMCPPs. The surface of the PWMCPP with a wire mesh volume fraction of 12.45% contains circular and irregular micropores (Figure 2a), and the metallurgical bonding between powder and powder was complete. The appearance of round micropores was due to the increase in metallurgical bonding degree between powder and powder with the increase of sintering temperature, and the irregular micropores gradually merge into round micropores. The surface pores of PWMCPP with a wire mesh diameter of 50 μm were mainly square pores and triangular pores (Figure 2b). Part of the square pores of wire mesh was retained, and the other part of the square pores was gradually fused into triangular pores due to rolling and a high-temperature sintering. When sintering temperature was 1160 °C, most of the wire mesh and wire mesh in PWMCPPs were in a clear separation state (Figure 2c). When the sintering temperature was 1310 °C, there was an obvious metallurgical interface between the wire mesh and wire mesh in PWMCPPs (Figure 2d). This is because higher sintering temperature was conducive to the growth and fusion of sintering neck, which increased the metallurgical bonding degree between the wire meshes [27].

### 3.2. Charpy Impact Properties of Novel PWMCPPs

#### 3.2.1. Effect of Wire Mesh Volume Fraction on Charpy Impact Properties

**Figure 3:**
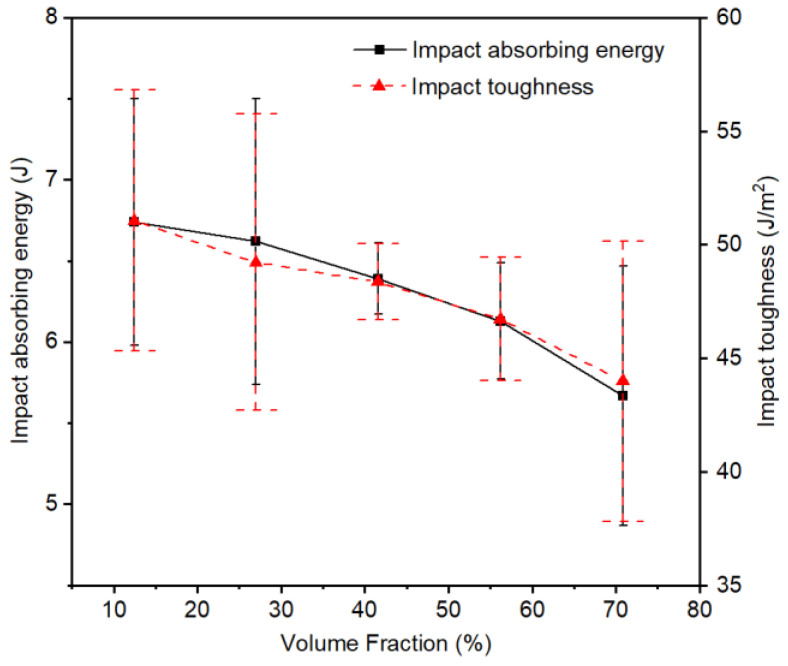
Variation trend of the impact properties of PWMCPPs with different wire mesh volume fractions.

The Charpy impact test results of PWMCPPs with different volume fractions of wire mesh are summarized in Table 2. The value of impact toughness was calculated from the value of impact absorbed energy according to Equation (3). Figure 3 shows that the absorption energy and impact toughness of PWMCPPs decreased with increasing wire mesh volume fraction. Specifically, when the volume fraction of wire mesh increased from 12.45% to 70.81%, the absorption energy of the PWMCPP decreased from 6.74 J to 5.67 J, with a reduction ratio of 15.88%, and the impact toughness decreased from 51.07 J/cm^2^ to 43.99 J/cm^2^, with a reduction ratio of 13.86%. Because the PWMCPP with a lower wire mesh volume fraction had more metallurgical bonding joints were used to bear the impact load, so it absorbed more impact energy and had better impact toughness. Because the impact test time was very short, the PWMCPP with higher volume content of wire mesh had no time to fully harden and further improve the mechanical properties under impact load, so it showed lower impact toughness. According to Figure 3, it can be seen that the variation trend of impact toughness of PWMCPPs with different wire mesh volume fractions has a large ranges of error bars. This is because the powder volume fractions of PWMCPPs with different wire mesh volume fractions were also different, and the pore structure formed by metallurgical bonding between powder and powder was more complex than that between wire mesh and wire mesh. As shown in Figure 2a, there are many irregular micropores in PWMCPPs with low wire mesh volume fractions. When the irregular micropores were subjected to the impact load, the stress situation was complex, and it was easier to produce cracks and damages. At the same time, the number of irregular micropores in different specimens with the same wire mesh volume fractions were also different, which further aggravated the instability of impact toughness.

#### 3.2.2. Effect of Wire Mesh Diameter on Charpy Impact Properties

**Figure 4:**
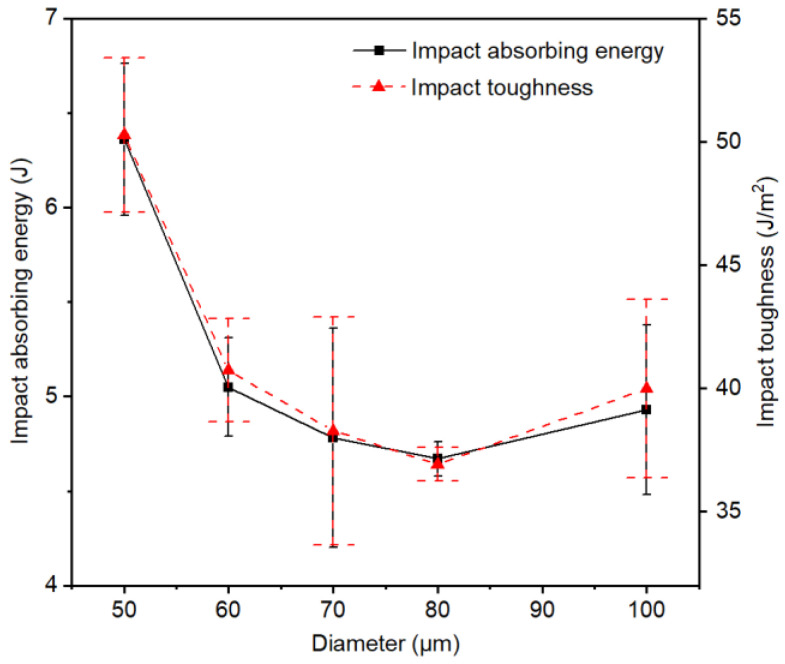
Variation trend of the impact properties of PWMCPPs with different wire mesh diameters.

Table 3 shows the Charpy impact test results of PWMCPPs with different wire mesh diameters. Figure 4 shows that the absorption energy and impact toughness of the PWMCPP decreased first and then increased with the increasing of wire mesh diameter. Specifically, when the diameter of the wire mesh increased from 50 μm to 80 μm, the absorption energy of the PWMCPP decreased from 6.36 J to 4.67 J, and the impact toughness decreased from 50.28 J/cm^2^ to 36.92 J/cm^2^, with a reduction ratio of 26.57%. Specifically, when the wire mesh diameter increased from 50 μm to 60 μm, the impact toughness decreased sharply. This is because with the increase of wire mesh diameter, the metallurgical interfaces between wire mesh layers decreased, and the internal pores increased. When the specimen was subjected to the impact load, it was easier to crack and damage. When the wire mesh diameter increased from 80 μm to 100 μm, the absorption power of the PWMCPP increased from 4.67 J to 4.93 J, with an increase ratio of 5.57%. Impact toughness increased from 36.92 J/cm^2^ to 39.99 J/cm^2^, with an increase ratio of 8.32%. This is because with the increase of the wire mesh diameter, the metallurgical interface between the mesh in unit volume decreased [28], and the structure available to bear the impact load was reduced, so that the stress of single metallurgical interface increased, which made the impact toughness of the PWMCPP decrease [29]. However, when the diameter of the wire mesh increased to a certain extent, the coarse wire mesh has a greater ability to bear the impact load than the fine wire mesh, so that the PWMCPP with larger mesh diameter had higher impact toughness. At the same time, according to Figure 4, it can be seen that the changes of wire mesh diameter had a minor effect on the impact toughness of the PWMCPPs.

#### 3.2.3. Effect of Sintering Temperature on Charpy Impact Properties

**Figure 5:**
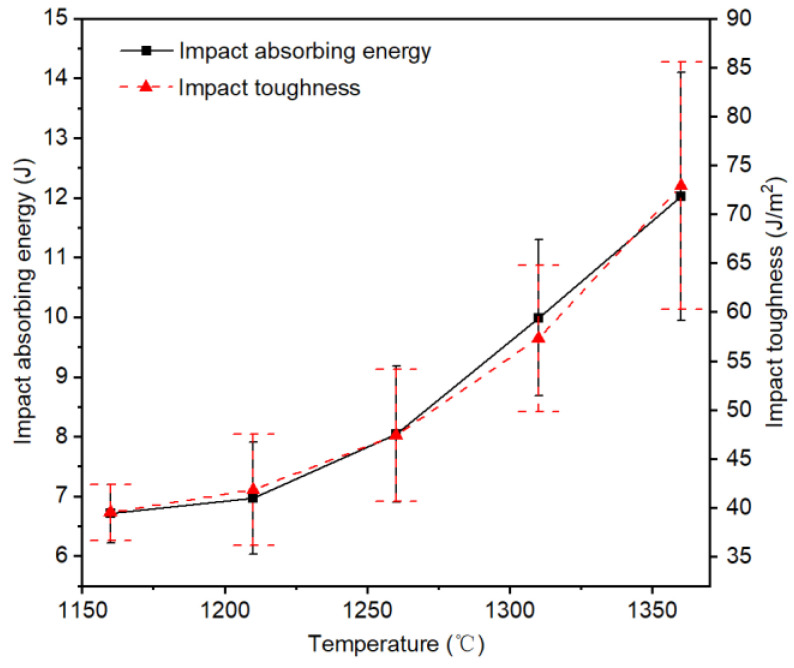
Variation trend of the impact properties of PWMCPPs with different sintering temperatures.

Table 4 shows the Charpy impact test results of PWMCPPs with a wire mesh diameter of 120 μm and different sintering temperatures. Figure 5 shows that the impact absorption energy and impact toughness of the PWMCPP increased with the increasing sintering temperature. Specifically, when the sintering temperature increased from 1160 °C to 1360 °C, the impact absorption energy of the PWMCPP increased from 6.71 J to 12.02 J, with an increased ratio of 79.14%, and the impact toughness increased from 39.54 J/cm^2^ to 72.95 J/cm^2^, with an increased ratio of 84.5%. This exceeds the impact toughness limit of porous twisted steel wire of 17.9 J/cm^2^, reported in the literature [26], exceeding it by 307.54%. This was because the increase in sintering temperature, on the one hand, accelerated the material migration movement [30], meaning the coarse sintering neck formed between powder and powder, powder and wire mesh, and the cross part of the wire mesh. On the other hand, higher temperature provided sufficient energy for molecular transition, which helped to increase the metallurgical combination between parallel distributed wire meshes [21]. Therefore, impact performance of the PWMCPP was improved effectively.

### 3.3. Charpy Impact Fracture Morphology

**Figure 6:**
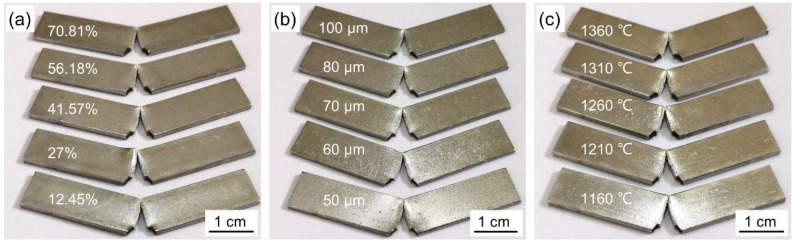
Macrograph of PWMCPPs after impact tests. (**a**) Different volume fractions; (**b**) different diameters; (**c**) different sintering temperatures.

Figure 6 shows the macrograph of PWMCPPs with different process factors: different wire mesh volume fractions (Figure 6a), different wire mesh diameters (Figure 6b), and different sintering temperatures (Figure 6c) after impact test. The powder layer and wire mesh layer in PWMCPPs were fractured after impact, and the cracks of the samples were produced from the V-notch, and gradually expanded in the opposite direction of the impact load direction. In the area around the crack, there is an obvious deformation and failure zone. The contact area between the middle part of the sample and the pendulum shows obvious arching phenomenon. This is because the pendulum has the maximum kinetic energy when it rushes down to the lowest point. Under the huge impact force of the pendulum, severe plastic deformation occurs in the area of the specimen in contact with the pendulum. There is an obvious concave phenomenon in the middle of the fracture, which indicates that the specimen was subjected to a strong tensile stress [31]. After impact test, there was no debris falling from the fracture surface, and the fracture surface shows obvious ductile fracture characteristics [32,33].

**Figure 7:**
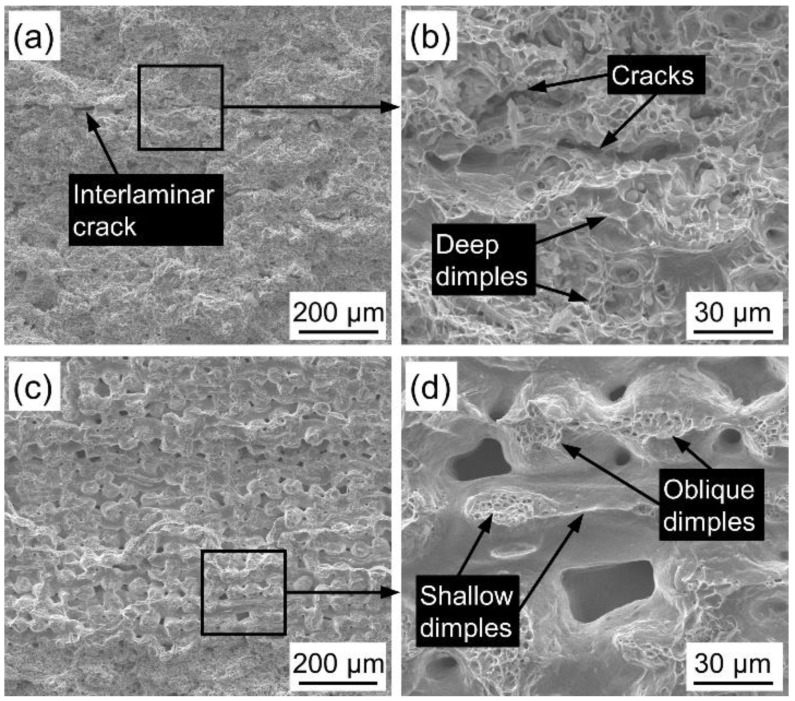
Impact fractures morphology of PWMCPPs with different wire mesh volume fractions. (**a**) Lower magnification and (**b**) higher magnification of the PWMCPP of 12.45% wire mesh volume fraction; (**c**) lower magnification and (**d**) higher magnification of the PWMCPP with 70.81% wire mesh volume fraction.

Figure 7 shows the fracture morphology of PWMCPPs with different wire mesh volume fractions after impact test. The fracture of the PWMCPP with wire mesh volume fraction of 12.45% has obvious interlaminar cracks (Figure 7a). The reason is that the bonding degree between layers was weaker than that between powders, and cracks are easy to occur in the weak layer [34]. Figure 7b is a partial enlarged view of Figure 7a. The fracture surface has obvious dimple structure (Figure 7b), which indicates that the fracture of PWMCPPs belongs to ductile fracture. The void content of the PWMCPP with 70.81% wire mesh volume fraction (Figure 7c) is higher than that of PWMCPP of 12.45% wire mesh volume fraction. Figure 7d is a partial enlarged view of Figure 7c. The main fracture forms are the metallurgical interface fracture between the wire mesh and the fractures of the wire mesh itself. Due to the impact load, dimples are mainly inclined dimples, which are opposite to the impact load direction (Figure 7d). There are few dimples between parallel meshes, and the dimples are shallow (Figure 7d). The number and depth of dimples of the PWMCPP of 12.45% wire mesh volume fraction are larger than those of the PWMCPP with 70.81% wire mesh volume fraction.

**Figure 8:**
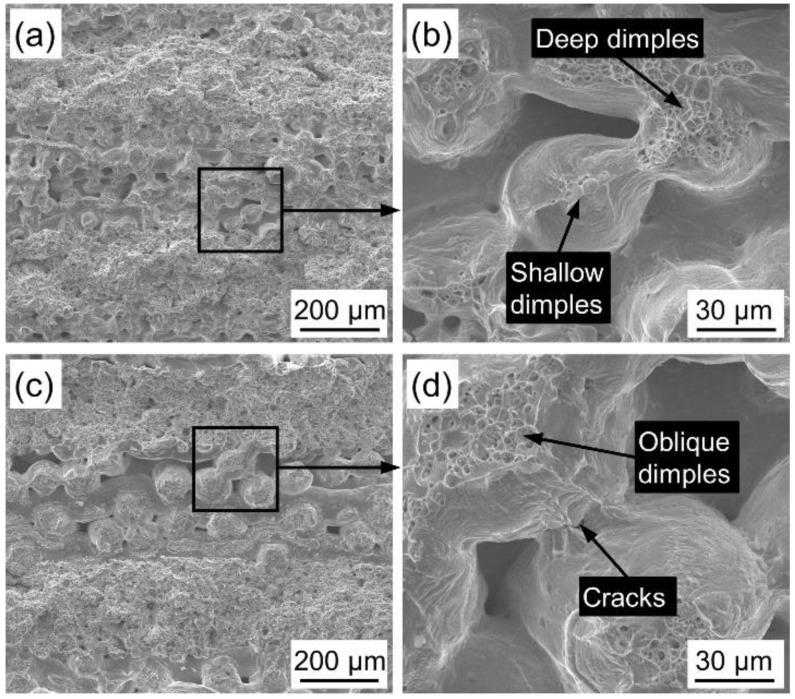
Impact fractures morphology of PWMCPPs with different wire mesh diameters. (**a**) Lower magnification and (**b**) higher magnification of the PWMCPP with 50 μm wire mesh diameter; (**c**) lower magnification and (**d**) higher magnification of the PWMCPP with 100 μm wire mesh diameter.

Figure 8 shows the fracture morphology of PWMCPPs with different wire mesh diameters after impact test. The void content of the PWMCPP with a wire mesh diameter of 50 μm (Figure 8a) is less than that of the PWMCPP with a wire mesh diameter of 100 μm (Figure 8c). Figure 8b is a partial enlarged view of Figure 8a. Wire mesh with more metallurgical bonding surface has deeper and more numerous dimples than single wire mesh after impact test (Figure 8b). The PWMCPP with wire mesh diameter of 50 μm has smaller pores, and more wire mesh bore impact load in the per unit area than in the PWMCPP with wire mesh diameter of 100 μm, which meant the PWMCPP with wire mesh diameter of 50 μm had higher impact toughness.

**Figure 9:**
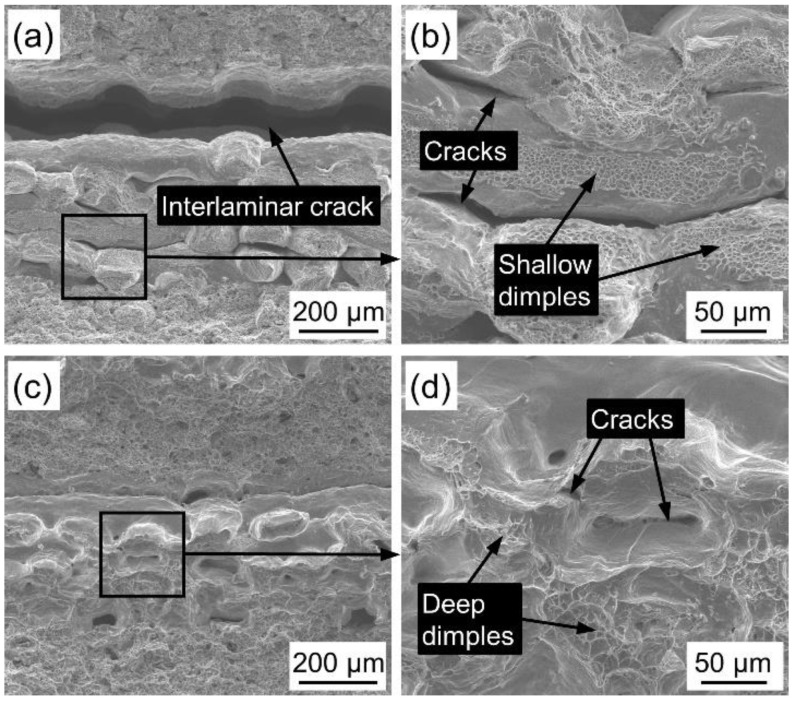
Impact fractures morphology of PWMCPPs with different sintering temperatures. (**a**) Lower magnification and (**b**) higher magnification of the PWMCPP with 1160 °C sintering temperature; (**c**) lower magnification and (**d**) higher magnification of the PWMCPP of 1360 °C sintering temperature.

Figure 9 shows the fracture morphology of PWMCPPs with different sintering temperatures after impact test. Fracture surface of the PWMCPP sintered at 1160 °C has a wide interlaminar crack (Figure 9a), which was due to the low sintering temperature and less effective metallurgical bonding surface formed between powder and wire mesh layers. Under the impact load, the metallurgical bonding surface breaks and cracks were formed due to the shear force between layers. Figure 9b is a partial enlarged view of Figure 9a. Under the impact load, there are obvious cracks between the wire mesh and wire mesh, and the dimple depth between the parallel wire mesh and wire mesh is shallow (Figure 9b). This is due to the low sintering temperature and the low metallurgical bonding degree between the wire mesh and wire mesh. Fracture surface of the PWMCPP sintered at 1360 °C has few pores and no interlaminar cracks (Figure 9c). There are obvious cracks on the metallurgical interface between wire mesh and wire mesh, and there are many deep dimples on the fracture surface (Figure 9d). This is due to the higher sintering temperature, the metallurgical bonding degree between wire mesh and powder was significantly improved. The sintering neck and the metallurgical bonding area were increased, and the plastic deformation ability of the material was improved, which is one of the important reasons for the improvement of impact toughness [35]. Under the impact load, cracks and deep dimples were formed due to the fracture of sintering neck and wire mesh.

## 4. Conclusions

(1) In this paper, a novel PWMCPP was fabricated by the composite rolling and vacuum solid-state sintering of 304 stainless steel powder and wire mesh as raw materials. SEM images show that PWMCPPs have different shapes and sizes of micropores.
(2) The effects of different wire mesh volume fractions, mesh diameters, and sintering temperatures on the Charpy impact properties of PWMCPPs were studied. The results show that the impact toughness of PWMCPPs decreases with increasing wire mesh volume fraction, and increases first and then decreases with increasing wire mesh diameter, and increases with increasing sintering temperature. Among them, the sintering temperature has the most obvious effect on the impact toughness of PWMCPPs. When the sintering temperature increased from 1160 °C to 1360 °C, the impact toughness increased from 39.54 J/cm^2^ to 72.95 J/cm^2^, with an increased ratio of 84.5%.
(3) The macroscopic image of PWMCPPs after impact testing shows a large plastic deformation, which indicates that PWMCPPs have an obvious ductile fracture mechanism.
(4) The mechanism of microscopic impact damage was mainly manifested as the tearing of metallurgical joint between powder and wire mesh layer, between powder and powder, and between wire mesh and wire mesh, and fracture of the wire mesh. Interlaminar cracks, tearing fractures, and oblique dimples were the main characteristics of impact fracture morphology of PWMCPPs.
(5) Low volume fraction and small diameter of wire mesh and sintering temperature over 1310 °C were beneficial for PWMCPPs to obtain higher impact toughness. Based on the porous structure and impact toughness of PWMCPPs, further research is planned on its application in energy absorption, vibration reduction, filtration, and other fields.

## Figures and Tables

**Table 1:** PWMCPPs with different processing parameters.

Volume Fraction (%)	Wire Mesh Diameter (μm)	Sintering Temperatures (°C)
12.45	40	1310
27	40	1310
41.57	40	1310
56.18	40	1310
70.81	40	1310
65.19	50	1310
65.17	60	1310
64.98	70	1310
65.57	80	1310
65.35	100	1310
65.7	120	1160
65.7	120	1210
65.7	120	1260
65.7	120	1310
65.7	120	1360

**Table 2:** Charpy impact properties of PWMCPPs with different wire mesh volume fractions.

Volume Fraction (%)	Thickness (mm)	Porosity (%)	Impact Absorbing Energy (J)	Impact Toughness (J/cm^2^)
12.45	1.65	5.47	6.74 ± 0.76	51.07 ± 5.75
27	1.68	5.59	6.62 ± 0.88	49.23 ± 6.53
41.57	1.65	5.82	6.39 ± 0.22	48.37 ± 1.67
56.18	1.64	6.01	6.13 ± 0.36	46.73 ± 2.72
70.81	1.61	6.22	5.67 ± 0.80	43.99 ± 6.18

**Table 3:** Charpy impact properties of PWMCPPs with different wire mesh diameters.

Wire Mesh Diameter (μm)	Thickness (mm)	Porosity (%)	Impact Absorbing Energy (J)	Impact Toughness (J/cm^2^)
50	1.58	7.26	6.36 ± 0.40	50.28 ± 3.13
60	1.55	7.51	5.05 ± 0.26	40.73 ± 2.10
70	1.56	7.62	4.78 ± 0.58	38.27 ± 4.63
80	1.58	7.96	4.67 ± 0.09	36.92 ± 0.68
100	1.54	8.21	4.93 ± 0.45	39.99 ± 3.63

**Table 4:** Charpy impact properties of PWMCPPs with different sintering temperatures.

Sintering Temperatures (°C)	Thickness (mm)	Porosity (%)	Impact Absorbing Energy (J)	Impact Toughness (J/cm^2^)
1160	2.12	6.46	6.71 ± 0.49	39.54 ± 2.86
1210	2.08	6.04	6.97 ± 0.94	41.87 ± 5.68
1260	2.12	5.82	8.04 ± 1.14	47.42 ± 6.74
1310	2.18	5.66	9.99 ± 1.31	57.30 ± 7.51
1360	2.06	5.41	12.02 ± 2.08	72.95 ± 12.65

## Data Availability

The data presented in this study are available on request from the corresponding author after obtaining permission of authorized person.
